# Supplementary material for: Pathophysiological Significance of Neutrophilic Transfer RNA-Derived Small RNAs in Asymptomatic Moyamoya Disease
Source: Cells. 2021 May 1;10(5):1086. doi: 10.3390/cells10051086 (PMC8147334; doi:10.3390/cells10051086)

2D Structure	Local AU	Position

```
398                               426
                      8mer-1a
 5'-aaaACTCTATGAATG-TAGTGAATGTGGGa-3' utr
       |  ||:  |:|  :|  |||    |||||||
 3'-uccUUAGG-AUUGGCGAUCUGGUACACCCu-5' tRF
                 16151413     7 6 5 4 3 2
              3' Pairing         Seed
```

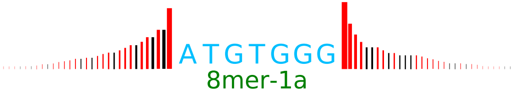

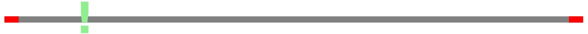

2D Structure	Local AU	Position

```
480                                510
                       8mer-1a
 5'-gaGAAACCCTATGAATGCACTGAATGTGGGa-3' utr
      |||  ||   |:    |:  ||:   |||||||
 3'-ucCUUAGGAUUGGCGAUCUGG--UACACCCu-5' tRF
               16151413       7 6 5 4 3 2
            3' Pairing          Seed
```

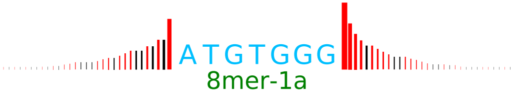

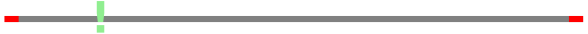

Supplement: Supplementary file 1 [file cells-10-01086-s001.zip › cells-1149161 3rd supplementary materials/Data Supplements/Data Supplement V.zip!/Data supplement 6-mRNAs targets of tsRNAs/tsRNA-Glu-TTC-010/Diagrams/tRF-Glu-TTC-010_vs_ENST00000437590(ZNF487).pdf]
